# International pooled study on diet and bladder cancer: the bladder cancer, epidemiology and nutritional determinants (BLEND) study: design and baseline characteristics

**DOI:** 10.1186/s13690-016-0140-1

**Published:** 2016-07-06

**Authors:** Maria E. Goossens, Fatima Isa, Maree Brinkman, David Mak, Raoul Reulen, Anke Wesselius, Simone Benhamou, Cristina Bosetti, Bas Bueno-de-Mesquita, Angela Carta, Md Farouk Allam, Klaus Golka, Eric J. Grant, Xuejuan Jiang, Kenneth C. Johnson, Margaret R. Karagas, Eliane Kellen, Carlo La Vecchia, Chih-Ming Lu, James Marshall, Kirsten Moysich, Hermann Pohlabeln, Stefano Porru, Gunnar Steineck, Marianne C. Stern, Li Tang, Jack A. Taylor, Piet van den Brandt, Paul J. Villeneuve, Kenji Wakai, Elisabete Weiderpass, Emily White, Alicja Wolk, Zuo-Feng Zhang, Frank Buntinx, Maurice P. Zeegers

**Affiliations:** Department of General Practice, Katholieke Universiteit Leuven, ACHG-KU Leuven, Kapucijnenvoer 33, Blok J, bus 7001, 3000 Leuven, Belgium; Department of Public Health, Epidemiology and Biostatistics, University of Birmingham, Birmingham, UK; The Cancer Council Victoria, Melbourne, Australia; NUTRIM School for Nutrition and Translational Research in Metabolism, University of Maastricht, Maastricht, The Netherlands; INSERM U946, Variabilite Genetique et Maladies Humaines, Fondation Jean Dausset / CEPH, Paris, France; Laboratory of General Epidemiology, Istituto di Ricerche Farmacologiche “Mario Negri”, Milan, Italy; Determinants of Chronic Diseases (DCD), National Institute for Public Health and the Environment (RIVM), Bilthoven, The Netherlands; Gastroenterology and Hepatology, University Medical Centre, Utrecht, The Netherlands; Epidemiology and Biostatistics, The School of Public Health, Imperial College London, London, UK; Social and Preventive Medicine, Faculty of Medicine, University of Malaya, Kuala Lumpur, Malaysia; Department of Medical and Surgical Specialties, Radiological Sciences and Public Health, Section of Public Health and Human Sciences, University of Brescia, Brescia, Italy; Department of Preventive Medicine and Public Health, Faculty of Medicine, University of Cordoba, Cordoba, Spain; Leibniz Research Centre for Working Environment and Human Factors at TU Dortmund, Dortmund, Germany; Department of Epidemiology Radiation Effects Research Foundation, Hiroshima, Japan; Department of Preventive Medicine, University of Southern California, Los Angeles, CA USA; Department of Epidemiology and Community Medicine, University of Ottawa, Ottawa, ON Canada; Department of Epidemiology, Geisel School of Medicine at Dartmouth, Hanover, NH USA; Leuven University Centre for Cancer Prevention (LUCK), Leuven, Belgium; Department of Clinical Medicine and Community Health, University of Milan, Milan, Italy; Department of Urology, Buddhist Dalin Tzu Chi General Hospital, Dalin Township, 62247 Chiayi County Taiwan; Department of Cancer Prevention and Control, Roswell Park Cancer Institute, Buffalo, NY USA; Leibniz Institute for Prevention Research and Epidemiology – BIPS, Bremen, Germany; Department of Oncology and Pathology, Division of Clinical Cancer Epidemiology, Karolinska Hospital, Stockholm, Sweden; Epidemiology Branch, and Epigenetic and Stem Cell Biology Laboratory, National Institute of Environmental Health Sciences, NIH, Research Triangle Park, NC USA; Department of Epidemiology, Schools for Oncology and Developmental Biology and Public Health and Primary Care, Maastricht University Medical Centre, Maastricht, The Netherlands; Population Studies Division Health Canada, Ottawa, ON Canada; Department of Preventive medicine, Nagoya University Graduate School of Medicine, Nagoya, Japan; Department of Medical Epidemiology and Biostatistics, Medical Epidemiology, Karolinska Institutet, Stockholm, Sweden; Department of Research, Cancer Registry of Norway, Institute of Population-Based Cancer Research, Oslo, Norway; Genetic Epidemiology Group, Folkhälsan Research Center, Helsinki, Finland; Department of Community Medicine, University of Tromsø, The Arctic University of Norway, Tromsø, Norway; Fred Hutchinson Cancer Research Center, Seattle, WA USA; Division of Nutritional Epidemiology, Institute of Environmental Medicine, Karolinska Institutet, Stockholm, Sweden; Departments of Epidemiology, UCLA Center for Environmental Genomics, Fielding School of Public Health, University of California, Los Angeles (UCLA), Los Angeles, CA USA; CAPHRI School for Public Health and Primary Care, University of Maastricht, Maastricht, The Netherlands; School of Cancer Sciences, University of Birmingham, Birmingham, UK

**Keywords:** Bladder cancer, Diet, Risk, Pooled analysis

## Abstract

**Background:**

In 2012, more than 400,000 urinary bladder cancer cases occurred worldwide, making it the 7^th^ most common type of cancer. Although many previous studies focused on the relationship between diet and bladder cancer, the evidence related to specific food items or nutrients that could be involved in the development of bladder cancer remains inconclusive. Dietary components can either be, or be activated into, potential carcinogens through metabolism, or act to prevent carcinogen damage.

**Methods/design:**

The BLadder cancer, Epidemiology and Nutritional Determinants (BLEND) study was set up with the purpose of collecting individual patient data from observational studies on diet and bladder cancer. In total, data from 11,261 bladder cancer cases and 675,532 non-cases from 18 case–control and 6 cohort studies from all over the world were included with the aim to investigate the association between individual food items, nutrients and dietary patterns and risk of developing bladder cancer.

**Discussion:**

The substantial number of cases included in this study will enable us to provide evidence with large statistical power, for dietary recommendations on the prevention of bladder cancer.

## Background

In 2012, more than 400,000 urinary bladder cancer (UBC) cases occurred worldwide, making it the 7th most common type of cancer [1]. Due to lifetime ongoing cystoscopies and recurrent treatment episodes, UBC is the most expensive malignancy in terms of healthcare expenditure in the USA and in most Western countries [2, 3]. The effect of diet in the prevention of UBC could be more pronounced compared to other types of cancer as dietary components are often excreted through the urine. Dietary components can either be, or be activated into, potential carcinogens through metabolism, or act to prevent carcinogen damage [4].

Although many previous studies focused on the relationship between diet and UBC, the evidence related to specific food items or nutrients that could be involved in the development of UBC remains inconclusive. The World Cancer Research Fund (WCRF) concluded in their most recent WCRF/AICR expert report [5] that there is some evidence for an decreased risk of bladder cancer with greater consumption of vegetables, fruit and tea and strong evidence that drinking water containing arsenic increases the risk of bladder cancer. A potential reason for the absence of evidence between specific foods and nutrients and the risk of UBC is that associations between cancer risk and dietary intake are usually weak and most previous studies may have had insufficient sample size and thus missed adequate statistical power for detailed analyses on individual food items, for subgroup analyses and for food-food interactions. Pooling of individual data of existing epidemiological studies on diet and UBC might therefore be an effective way to increase the current knowledge on the influences of foods, nutrients and dietary patterns on UBC risk. The influence of occupational risk and pollutants in the water, such as arsenic, are not part of this investigation. Occupational risk factors were identified as risk factors for bladder cancer [6]. However, as the frequency of having a high-risk occupation is very low (<3 %) this could not importantly confound the results. For this reason, the BLEND study as well as most previous bladder cancer epidemiological studies have not corrected for occupation in their analyses.

Within the BLadder cancer, Epidemiology and Nutritional Determinants (BLEND) study, we aim to investigate comprehensively the association between individual food items, nutrients, and dietary patterns and risk of developing UBC. The results of this study will likely aid in developing and reviewing current dietary recommendations for the prevention of UBC. In this paper we report on the methodology and baseline characteristics of the BLEND study.

## Methods/design

### Included epidemiological studies

Possible eligible epidemiological studies reporting on diet and UBC have been identified by a computerized search of Medline (National Library of Medicine, Bethesda, Maryland) (1966-Sept 2009), and Embase (Elsevier B. V., Amderstam, the Netherlands (1974-Sept 2009) using the medical subject headings (MeSH; National Library of Medicine, Bethesda, Maryland) “urinary bladder neoplasms” and “risk” and the free-text word “risk”. The search was restricted to the MeSH term “humans”. All articles from peer-reviewed journals, reporting on the association between diet and risk of UBC were selected. Within these articles, we identified the eligible studies that used a case–control or a cohort design, had data on diet and a minimum number of cases of 40 patients. The principal investigators of these eligible studies were contacted and invited to participate in our collaborative project. There was no restriction about the amount of available diet items, however, data on confounders, especially, smoking, had to be available.

### Data harmonization

To harmonize our data, a common codebook was created based on the Eurocode 2 Core classification version 99/2 [7]. The Eurocode 2 Food Coding System was originally developed to serve as a standard instrument for nutritional surveys in Europe and to serve the need for food intake comparisons within the European FLAIR Eurofoods-Enfant Project [8]. The Eurocode 2 classification System unambiguously defines which types of food are covered or not within each food category so that the potential for misclassification is limited. The System provides coding for food items consumed all over the world. Coding has been done centrally by the researchers of the Blend team. One part of the team did the coding, while the other part of the team checked for possible errors. Translation of the questionnaires in English was provided by the principle investigator for studies in other languages. Apart from the variables on diet, we collected non-dietary data such as, study design, age, gender, ethnic group, TNM Classification of Malignant Tumors (TNM), smoking status, smoking frequency and duration, and family history. Each participant was assigned a random and unique identification number. Analyses were restricted to adults, i.e. participants younger than 18 years were excluded. Categorical data have been checked by producing frequency tables to identify inaccurate coding while continuous data have been checked performing descriptive statistics. Possible coding errors and missing data within the provided data of each study were discussed with the principal investigator and updated accordingly. Outliers, defined as values outside the general distribution of the data, were identified after visual inspection of the resultant scatterplots and omitted [9].

### Baseline characteristics

**Fig. 1:**
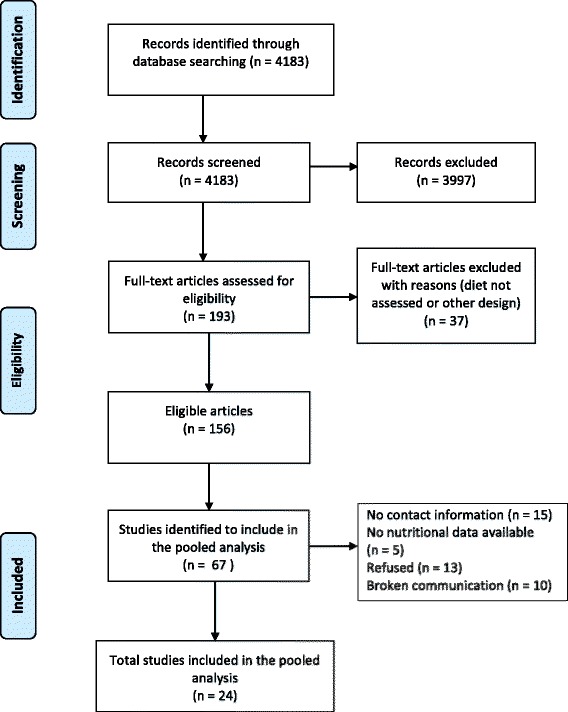
Flow diagram of the Bladder cancer Epidemiology and Nutritional Determinants study (BLEND)

In total 67 potentially eligible studies from 156 retrieved articles were identified (Fig. 1). Thirty-eight investigators agreed to participate and 24 [10–34] provided data (Table 1). Reasons for non-participation after initially agreement were: no data on diet or the minimum set of confounders available, the workload that was already too high and the wish to publish the results on nutrition first before participating in a pooled study. With some investigators, we lost communication after initial contact. The first datasets and codebooks were collected in March 2009 while the last dataset was included in March 2016. Another two new studies, one case–control and one cohort study are available for inclusion.

**Table 1:** Characteristics of the studies included in the pooled analysis of the Bladder cancer Epidemiology and Nutritional Determinants study (BLEND)

Study	Country	Recruitment period	Study design	Cases *N*			Controls *N*		
				men	women	total	men	women	total
Case–control studies									
Los-Angeles bladder cancer Case–control study [10]	USA	1987–1999	Population based case–control	1,307	353	1,660	1,237	349	1,586
Roswell Park Cancer Institute [11]	USA	1982–1998	Hospital-based case–control	164	53	217	501	163	664
Belgian Case–control study on bladder cancer [12]	Belgium	1999–2004	Population based case–control	172	28	200	228	156	384
Aichi Prefecture Case–control study [13]	Japan	1996–1999	Hospital-based case–control	245	58	303	244	59	303
Kaohsiung [14]	Taiwan	1996–1997	Hospital-based case–control	31	9	40	124	36	160
Hessen Case–control study on bladder cancer [15]	Germany	1989–1992	Hospital-based case–control	239	61	300	239	61	300
Stockholm Case–control study [16]	Sweden	1985–1987	Population based case–control	204	67	271	281	268	549
Roswell Park Memorial Institute Case–control study on bladder cancer [17]	USA	1957–1965	Hospital-based case–control	415	138	553	3,253	4,636	7,889
Reina Sofia University Hospital [18]	Spain	1997	Hospital-based case–control	74	11	85	89	41	130
New Hampshire bladder cancer study [19]	USA	1994–2001	Population based case–control	286	104	390	185	138	323
Italian Case–control study on bladder cancer [20]	Italy	1985–1993	Hospital-based case–control	617	110	727	766	298	1,064
Brescia bladder cancer study [21]	Italy	1997–2000	Hospital-based case–control	200	0	200	214	0	214
Dortmund Hörde study [22]	Germany	2009–2010	Hospital based case–control	145	48	193	177	56	233
National Enhanced Cancer Surveillance System (NESCC) [23]	Canada	1994–1997	Population based case–control	600	311	911	2,451	2,423	4,874
French INSERM study [24]	France	1984–1987	Hospital-based case–control	166	33	199	275	47	322
South and East China Case–control study on bladder and prostate cancer [25]	China	2005–2008	Hospital-based case–control	390	93	483	364	100	464
Molecular Epidemiology of Bladder Cancer and Prostate Cancer [26]	USA	1993–1997	Hospital-based case–control	149	45	194	243	58	301
North Carolina case control study [27]	USA	1987–1991	Hospital-based case–control	188	56	244	174	41	215
Cohort studies									
Swedish Mammography Cohort (SMC) & the Cohort of Swedish Men [28]	Sweden	1987–1990	Population based cohort	538	119	657	2,188	484	2,672
Netherlands Cohort Study on diet and cancer [29]	The Netherlands	1986–2003	Population based cohort	779	161	940	2,273	2,419	4,692
Women's Lifestyle and Health Study [30]	Norway, Sweden	1991–2006	Population based cohort	0	49	49	0	48,942	48,942
RERF atomic bomb survivors Study [31]	Japan	1950–2000	Population based cohort	216	85	301	19,362	28,249	47,611
VITamins and Lifestyle Study (VITAL) [32]	USA	2000–2008	Population based cohort	338	106	444	36,454	39,983	76,437
European Prospective Investigation into Cancer and Nutrition (EPIC) [33, 34]	Europe	1993–2006	Population based cohort	1,227	525	1,752	141,872	333,279	475,151
TOTAL	–	–	–	8,657	2,604	11,313	213,227	462,305	675,480

More than 2/3 of the case–control studies [11, 13–15, 17, 18, 20–22, 24–27] had a hospital-based case–control design. Ten studies [12, 16, 19–21, 24–28] were also part of the International Bladder Cancer Consortium that was formed in 2005 as an open scientific forum for genetic-epidemiologic researchers in the field of UBC. Most of the studies [12, 15, 16, 18, 20–22, 24, 28–30, 33, 34] were from Europe, eight studies [10, 11, 17, 19, 23, 26, 27, 32] were from the USA and Canada, and four [13, 14, 25, 31] studies were from Asia.

After excluding participants with unknown age (*n* = 5), unknown case–control status (*n* = 214) and unknown smoking status (*n* = 14,028) data of 686,793 participants were available for analyses of which 11,261 cases and 675,532 non-cases. The Brescia bladder cancer study [21] contained only male participants, while the Women’s Lifestyle and Health study consisted of only female participants. Most of the cases were from America to Europe while only 10 % were from Asia.

The cases of the European and Asian case–control studies had the highest male/female ratio (4:1) while their overall male/female ratio was 3:1 (Table 2). In general, controls were younger than cases, 57.0 versus 61.6 years and 51.8 versus 61.1 years, respectively for case–control studies and cohort studies with an exception for the Asian case–control studies (66.1 versus 64.9 years). Most of the participants were Caucasian, whereas only 10 % of the cases were Asian. In contrast with Asia, where one third of the cases were never smoker, only one fifth of the cases never smoked in Europe and USA. Overall, 40 % of the cases were smokers. Controls had significant less current and more never smokers than cases. For cohort studies, nearly half of the controls never smoked. Staging was not reported in 60 and 70 % respectively for the case–control and cohort studies.

**Table 2:** Characteristics of the study population of the Bladder cancer Epidemiology and Nutritional Determinants study (BLEND)

			Total				Europe				America				Asia	
	Cases		Controls		Cases		Controls		Cases		Controls		Cases		Controls	
	*N*	(()	*N*	(%)	*N*	(%)	*N*	(%)	*N*	(%)	*N*	(%)	*N*	(%)	*N*	(%)
Case–control studies																
*Gender*																
Male	5,592	(77.9)	11,045	(55.9)	1,817	(83.5)	2,269	(71.0)	3,109	(74.6)	8,044	(50.7)	666	(80.6)	732	(79.0)
Female	1,578	(22.1)	8,930	(44.1)	358	(16.5)	927	(29.0)	1,060	(25.4)	7,808	(49.3)	160	(19.4)	195	(21.0)
*Age* (mean, SD)	61.6	(11.4)	57.0	(14.4)	65.4	(9.7)	63.3	(10.9)	59.0	(11.1)	55.3	(14.5)	64.9	(13.1)	66.1	(12.2)
< 50	961	(13.4)	5,665	(28.4)	135	(6.2)	308	(9.6)	723	(17.3)	5,271	(33.3)	103	(12.5)	86	(9.3)
50– 59	1,832	(25.6)	4,501	(22.5)	407	(18.7)	787	(24.6)	1,261	(30.2)	3,549	(22.4)	164	(19.9)	165	(17.8)
60–64	1,399	(19.5)	2,772	(13.9)	381	(17.5)	555	(17.4)	929	(22.3)	2,1	(13.2)	89	(10.8)	117	(12.6)
65–69	1,122	(15.6)	2,842	(14.2)	482	(22.2)	567	(17.7)	522	(12.5)	2,138	(13.5)	118	(14.3)	137	(14.8)
≥ 70	1,856	(25.9)	4,195	(21.0)	770	(35.4)	979	(30.6)	734	(17.6)	2,794	(17.6)	352	(42.6)	422	(45.5)
*Ethnic group*																
Caucasian	4,438	(61.9)	15,057	(75.4)	593	(27.3)	831	(26.0)	3,845	(92.2)	14,226	(89.2)	782	(94.7)	767	(82.7)
Mixed	9	(0.1)	10	(0.1)	–	–	–	–	9	(0.2)	10	(0.1)	–	–	–	–
Asian	788	(11.0)	895	(4.5)	–	–	–	–	6	(0.1)	128	(0.8)	–	–	–	–
Black	52	(0.7)	748	(3.7)	–	–	–	–	52	(1.2)	748	(4.7)	–	–	–	–
Any other ethnic group	64	(0.9)	232	(1.2)	–	–	–	–	21	(0.5)	72	(0.5)	43	(5.2)	160	(17.3)
Unknown	1,819	(25.4)	3,033	(15.2)	1,582	(72.7)	2,365	(74.0)	236	(5.7)	668	(4.2)	1	(0.1)	–	–
*Tobacco smoking status*																
Current smoker	2,95	(41.1)	6,98	(34.9)	1,038	(47.7)	1,022	(32.0)	1,564	(37.5)	5,695	(35.9)	348	(42.1)	263	(28.4)
Former smoker	2,703	(37.7)	5,269	(26.4)	747	(34.3)	1,025	(32.1)	1,731	(41.5)	3,943	(24.9)	225	(27.2)	301	(32.5)
Never smoker	1,517	(21.2)	7,726	(38.7)	390	(17.9)	1,149	(36.0)	874	(21.0)	6,214	(39.2)	253	(30.6)	363	(39.2)
*Staging*																
Non–invasive	2,246	(31.3)	–	–	511	(23.5)	–	–	1,606	(38.5)	–	–	129	(15.6)	–	–
Invasive	609	(8.5)	–	–	73	(3.4)	–	–	366	(8.8)	–	–	170	(20.6)	–	–
Unknown	4,315	(60.2)	–	–	1,591	(73.1)	–	–	2,197	(52.7)	–	–	527	(63.8)	–	–
*Continent*																
Europe	2,175	(30.3)	3,196	(16.0)	–	–	–	–	–	–	–	–	–	–	–	–
America	4,169	(58.1)	15,852	(79.4)	–	–	–	–	–	–	–	–	–	–	–	–
Asia	826	(11.5)	927	(4.6)	–	–	–	–	–	–	–	–	–	–	–	–
Cohort studies																
*Gender*																
Male	2,866	(69.2)	205,678	(31.4)	2,544	(74.9)	146,333	(27.5)	338	(76.1)	39,983	(52.3)	216	(71.8)	19,362	(40.7)
Female	1,277	(30.8)	449,827	(68.6)	854	(25.1)	385,124	(72.5)	106	(23.9)	36,454	(47.7)	85	(28.2)	28,249	(59.3)
*Age* (mean, SD)	61.1	(8.5)	51.8	(10.8)	60.9	(7.9)	50.4	(10.2)	66.4	(6.4)	61.4	(7.4)	55.7	(12.0)	52.0	(13.6)
< 50	380	(9.2)	270,949	(41.3)	277	(8.2)	249,151	(46.9)	–	–	–	–	103	(34.2)	21,798	(45.8)
50–59	1,305	(31.5)	232,316	(35.4)	1,154	(34.0)	184,999	(34.8)	69	(15.5)	35,193	(46.0)	82	(27.2)	12,124	(25.5)
60–64	1,114	(26.9)	81,842	(12.5)	974	(28.7)	62,868	(11.8)	92	(20.7)	13,923	(18.2)	48	(15.9)	5,051	(10.6)
65–69	757	(18.3)	39,082	(6.0)	610	(18.0)	22,425	(4.2)	109	(24.5)	12,561	(16.4)	38	(12.6)	4,096	(8.6)
≥ 70	587	(14.2)	31,316	(4.8)	383	(11.3)	12,014	(2.3)	174	(39.2)	14,760	(19.3)	30	(10.0)	4,542	(9.5)
*Ethnic group*																
Caucasian	3,815	(92.1)	602,416	(91.9)	3,398	(100)	531,457	(100)	417	(93.9)	70,959	(92.8)	–	–	–	–
Asian	314	(7.6)	50,651	(7.7)	–	–	–	–	13	(2.9)	3,04	(4.0)	301	(100)	47,611	(100)
Black	7	(0.2)	969	(0.1)	–	–	–	–	7	(1.6)	969	1.3)	–	–	–	–
Any other ethnic group	1	(0.0)	475	(0.1)	–	–	–	–	1	(0.2)	475	(0.6)	–	–	–	–
Unknown	6	(0.1)	994	(0.2)	–	–	–	–	6	(1.4)	994	(1.3)	–	–	–	–
*Tobacco smoking status*																
Current smoker	1,677	(40.5)	156,467	(23.9)	1,418	(41.7)	130,871	(24.6)	61	(13.7)	6,411	(8.4)	198	(65.8)	19,185	(40.3)
Former smoker	1,594	(38.5)	185,006	(28.2)	1,296	(38.1)	149,472	(28.1)	280	(63.1)	33,651	(44.0)	18	(6.0)	1,883	(4.0)
Never smoker	872	(21.0)	314,032	(47.9)	684	(20.1)	251,114	(47.3)	103	(23.2)	36,375	47.6)	85	(28.2)	26,543	(55.7)
*Staging*																
Non–invasive	1,196	(28.9)	–	–	1,196	(35.2)	–	–	–	–	–	–	–	–	–	–
Invasive	661	(16.0)	–	–	661	(19.5)	–	–	–	–	–	–	–	–	–	–
Unknown	2,286	(55.2)	–	–	1,541	(45.4)	–	–	444	(100)	–	–	301	(100)	–	–
*Continent*																
Europe	3,398	(82.0)	531,457	(81.1)	–	–	–	–	–	–	–	–	–	–	–	–
America	444	(10.7)	76,437	(11.7)	–	–	–	–	–	–	–	–	–	–	–	–
Asia	301	(7.3)	47,611	(7.3)	–	–	–	–	–	–	–	–	–	–	–	–

Although all of the studies used a food frequency questionnaire (FFQ), the number of food items assessed varied widely (Table 3). Two studies [22, 24] only asked three and two specific items (beer, coffee and decaffeinated coffee), while others assessed dietary intake in more detail (from 9 [27] to 788 food items [12]). The mean number of food items per questionnaire was 107 and 132 after exclusion of those studies that reported only on beverages [14, 22, 24]. Most studies with a FFQ of more than 40 items had detailed information on dietary intake of meat, vegetables, fruit and beverages. The use of a validated FFQ questionnaire was reported in eight studies [12, 19, 23, 28–30, 32–34], while one study checked the reproducibility of its FFQ [20]. Most of the studies assessed portion size, while four studies [12, 28, 29, 33, 34] reported the quantitative intake of food items in grams. Six studies [10, 19, 28, 30, 32–34] also provided data on nutrients.

**Table 3:** Number of food items and portion size reported by each study within the Bladder cancer Epidemiology and Nutritional Determinants study (BLEND)

Study	Food items (*n*)	Portion size
Case–control studies		
Los-Angeles bladder cancer Case–control study [10]	49	Yes
Roswell Park Cancer Institute [11]	44	Yes
Belgian Case–control study on bladder cancer [12]	788	Yes
Aichi Prefecture Case–control study [13]	107	Yes
Kaohsiung [14]	41	Yes
Hessen Case–control study on bladder cancer [15]	26	No
Stockholm Case–control study [16]	188	Yes
Roswell Park Memorial Institute Case–control study on bladder cancer [17]	64	Yes
Reina Sofia University [18]	17	No
New Hampshire bladder cancer study [19]	121	Yes
Italian Case–control study on bladder cancer [20]	21	No
Brescia bladder cancer study [21]	40	Yes
Dortmund Hörde study [22]	3	Yes
National Enhanced Cancer Surveillance System (NESCC) [23]	69	Yes
French INSERM study [24]	2	No
South and East China Case–control study on bladder and prostate cancer [25]	52	No
Molecular Epidemiology of Bladder Cancer and Prostate Cancer [26]	90	Yes
North Carolina case control study [27]	9	No
Cohort studies		
Swedish Mammography Cohort (SMC) & the Cohort of Swedish Men [28]	96	No
Netherlands Cohort Study on diet and cancer, the Netherlands, 1986–2003 [29]	150	Yes
Women’s Lifestyle and Health Study [30]	98	Yes
RERF atomic bomb survivors Study [31]	102	No
Vital study [32]	126	Yes
European Prospective Investigation into Cancer and Nutrition (EPIC) [33, 34]	260^a^	Yes

^a^Dietary intake was assessed by a number of different instruments in the participating countries and the number of different food items varied from 88 (Norway) to 2443 (Sweden)

The consumption of beverages was reported in all the eighteen case–control studies. Five case–control studies [12, 13, 16, 19, 26] had detailed information for each of the larger food categories of the Eurocode 2 Food Coding System, while three studies [11, 23, 25] missed only data on sugar and/or fat (Table 4). Fat, grains, nuts and sugar were also missing in another four studies [10, 15, 17, 20]. The six cohort studies [28–34] had detailed information in each food categories with the exception of the RERF atomic bomb survivors study [31] which had no data on sugar intake.

**Table 4:** Numbers of cases and controls available for each food category included in the Bladder cancer Epidemiology and Nutritional Determinants study (BLEND)

	All Countries				Europe				America				Asia			
	Men		Women		Men		Women		Men		Women		Men		Women	
Food category (number of studies)	Ca *N*	Co *N*	Ca *N*	Co *N*	Ca *N*	Co *N*	Ca *N*	Co *N*	Ca *N*	Co *N*	Ca *N*	Co *N*	Ca *N*	Co *N*	Ca *N*	Co *N*
Case–control studies																
Milk and milk products (13) [10–17, 19, 20, 23, 25, 26]	4,734	9,251	1,388	7,442	1,231	1,514	266	783	2,838	7,005	962	6,464	665	732	160	195
Eggs and eggs products (11) [10–13, 15, 16, 19, 20, 23, 25, 26]	4,299	6,531	1,255	3,974	1,230	1,512	265	781	2,436	4,141	839	3,034	633	605	151	159
Meat and meat products (12) [10–13, 15–17, 19, 20, 23, 25, 26]	4,699	9235	1,377	7,716	1,231	1,513	265	783	2,833	7,114	961	6,774	635	608	151	159
Fish and fish products (11) [11–13, 15–17, 19, 20, 23, 25, 26]	3,197	7,391	960	7,144	1,229	1,511	265	781	1,335	5,275	544	6,204	633	605	151	159
Fats and oils (7) [10–13, 16, 19, 26]	2,299	2,292	634	984	371	500	94	419	1,689	1,559	484	506	239	233	56	59
Grain and grain products (11) [10–13, 16, 17, 19, 21, 23, 25, 26]	4,050	8,481	1,209	7,404	574	721	94	424	2,841	7,153	964	6,821	635	607	151	159
Pulses, seeds and nut products (8) [11–13, 16, 19, 23, 25, 26]	2,108	4,255	715	3,270	371	499	94	421	1,106	3,151	470	2,690	631	605	151	159
Vegetables (13) [10–13, 15–17, 19–21, 23, 25, 26]	4,942	10,086	1,403	8,648	1,429	1,727	265	783	2,881	7,754	987	7,706	632	605	151	159
Fruit and fruit products (13) [10–13, 15–17, 19–21, 23, 25, 26]	4,860	9,307	1,376	7,615	1,414	1,713	265	781	2,814	6,989	960	6,675	632	605	151	159
Sugar products (7) [12, 13, 16, 18, 19, 23, 26]	1,613	3,438	582	3,020	446	591	105	463	935	2,615	421	2,499	232	232	56	58
Beverages (18) [10–27]	5,509	10193	1,538	7,640	1,814	2,269	357	926	3,030	7,192	1,021	6,519	665	732	160	195
Cohort studies																
Milk and milk products (6) [28–34]	2,615	184,424	1,159	422,716	2,495	146,183	835	384,864	86	35,753	314	33,742	34	2,488	10	4,110
Eggs and eggs products (6) [28–34]	2,585	184,284	1,147	421,392	2,465	146,039	823	383,535	86	35,753	314	33,742	34	2,492	10	4,115
Meat and meat products (6) [28–34]	2,614	184,420	1,156	422,122	2,494	146,171	832	384,262	86	35,753	314	33,742	34	2,496	10	4,118
Fish and fish products (6) [28–34]	2,613	184,406	1,157	421,976	2,493	146,157	833	384,116	86	35,753	314	33,742	34	2,496	10	4,118
Fats and oils (6) [28–34]	2,527	181,544	1,130	421,335	2,420	146,029	810	384,710	86	35,753	314	33,742	21	1,762	6	2,883
Grain and grain products (6) [28–34]	2,618	184,446	1,158	422,738	2,498	146,194	834	384,876	86	35,753	314	33,742	34	24,99	10	4,120
Pulses, seeds and nut products (6) [28–34]	2,563	184,228	1,143	420,368	2,443	145,984	819	382,512	86	35,753	314	33,742	34	2,491	10	4,114
Vegetables (6) [28–34]	2,616	184,432	1,157	422,236	2,496	146,184	833	384,376	86	35,753	314	33,742	34	2495	10	4118
Fruit and fruit products (6) [28–34]	2,607	184,416	1,155	421,526	2,487	146,170	831	383,666	86	35,753	314	33,742	34	2493	10	4118
Sugar products (5) [28–30, 32–34]	2,556	181,860	1,143	417,615	2,470	146,107	829	383,873	86	35,753	314	33,742	0	0	0	0
Beverages (6) [28–34]	2,630	187,445	1,172	424,778	2,497	146,190	835	384,868	99	38,760	327	35,793	34	2495	10	4117

*Abbreviations*: *Ca* cases, *Co* controls, *N* number

## Discussion

The high number of cases (11,261) and controls (675,532) from 24 epidemiological studies included in the BLEND study makes the BLEND study the largest dataset on diet and UBC worldwide. A large sample size provides the potential to analyze in more detail food items rarely consumed [35] and allows delineating the generally weak association between UBC cancer and dietary intake for food categories. The advantage of pooling individual data compared to meta-analysis of aggregate data are multiple: it increases the power to detect the effect for food items more rarely consumed, it allows to adjust for the same confounding factors, gender, age, and smoking status, to test for interaction and to perform subgroup analyses [36, 37].

Demographic data in the BLEND study are consistent with the IARC CancerBase [1]. The male/female ratio in our dataset was 3:1. Worldwide the male/female ratio is 3.3:1. Europe is responsible for nearly 40 % of the UBC cases worldwide while the Asian population account for 28 % of the UBC incidence [1]. In our dataset, 49 % of the cases are from Europe while only 10 % of the cases are from Asia. The African and the Eastern Mediterranean region is responsible for only 9 % of the UBC incidence worldwide [1]. These regions are not represented in our dataset. In America and Europe, more than 90 % of the UBC cases are transitional cell carcinoma (TCC), while in Africa, up tot 40 % of the UBC cases can be squamous cell carcinomas (SCC) [38, 39] due to infection with Schistosoma haematobium (Bilharziasis) [40]. The Egyptian multi-center case–control study [41] had not yet been published when we collected our data. So, pooling of the data of the different countries is possible because most industrialized countries are likely to share the same risk factors for UBC. Otherwise, it will be possible to stratify analyses by region given the large number of included participants. We aim to update the BLEND database in the future with new available studies.

## Conclusion

The available data in the very large BLEND database will allow us to test associations between individual food items of the different food items categories, even those less commonly consumed, and the risk for UBC. We will also investigate food patterns such as the Mediterranean diet and the influence of nutrients on the risk of UBC. In addition, the large sample size will allow subgroup analyses.

## Abbreviations

BLEND, the BLadder cancer, Epidemiology, and Nutritional Determinants study; FFQ, food frequency questionnaire; OR, odds ratio; SCC, squamous cell carcinoma; SD, standard deviation; TCC, transitional cell carcinoma; TNM, TNM Classification of Malignant Tumors; UBC, urinary bladder cancer; WCRF, World Cancer Research Fund.
